# Morphotype induced changes in the life history and population dynamics of an hippolytid shrimp

**DOI:** 10.1038/s41598-023-47834-x

**Published:** 2023-11-23

**Authors:** Chryssa Anastasiadou, Roman Liasko, Ioannis Leonardos

**Affiliations:** 1 Fisheries Research Institute, Hellenic Agricultural Organization, 64007 Nea Peramos, Kavala, Greece; 2 Department of Biological Applications and Technology, University of Ioannina, University Campus, 45110 Ioannina, Greece

**Keywords:** Evolution, Zoology

## Abstract

One of the most exceptional, loud paradigm of shape polymorphisms constitutes the “rostral loss” condition in hippolytid shrimps. The intertidal shrimp *Hippolyte sapphica* includes two conspecific morphotypes, one of which demonstrates a neotenic rostrum (morph-B). Morphs’ rostral elongation is controlled by a single genetic locus, with long rostra (morph-A) representing the recessive state and short, larval-like ones the completely dominant state. Geometrics morphometry on the species morphotypes revealed also the homozygous/heterozygous state of the gene site along with some induced body’ adaptations, which compete the micro-evolutionary disadvantage of the “rostral loss”. We found recently that females’ viability and maternal energy investment selectively favors morph-A. The present contribution detects and discuss comparatively demographic and reproductive traits in species mixed (both morphs) and unmixed populations. Our results show that this sharp dimorphic rostral condition is a sex-related marker and that the species is gonochoric. Presence of morph-B results to (a) lower egg production (b) higher seasonally males’ percentage (c) morph-A females’ earlier maturation and (d) higher fecundity in morph-A mixed populations. It seems that the “rostral loss” state induces complex adaptations between the two morphotypes through sex ratios equilibria, morphotypes’ growth rates, and morphs’ fecundity differentiate inputs throughout the seasons.

## Introduction

**Figure 1:**
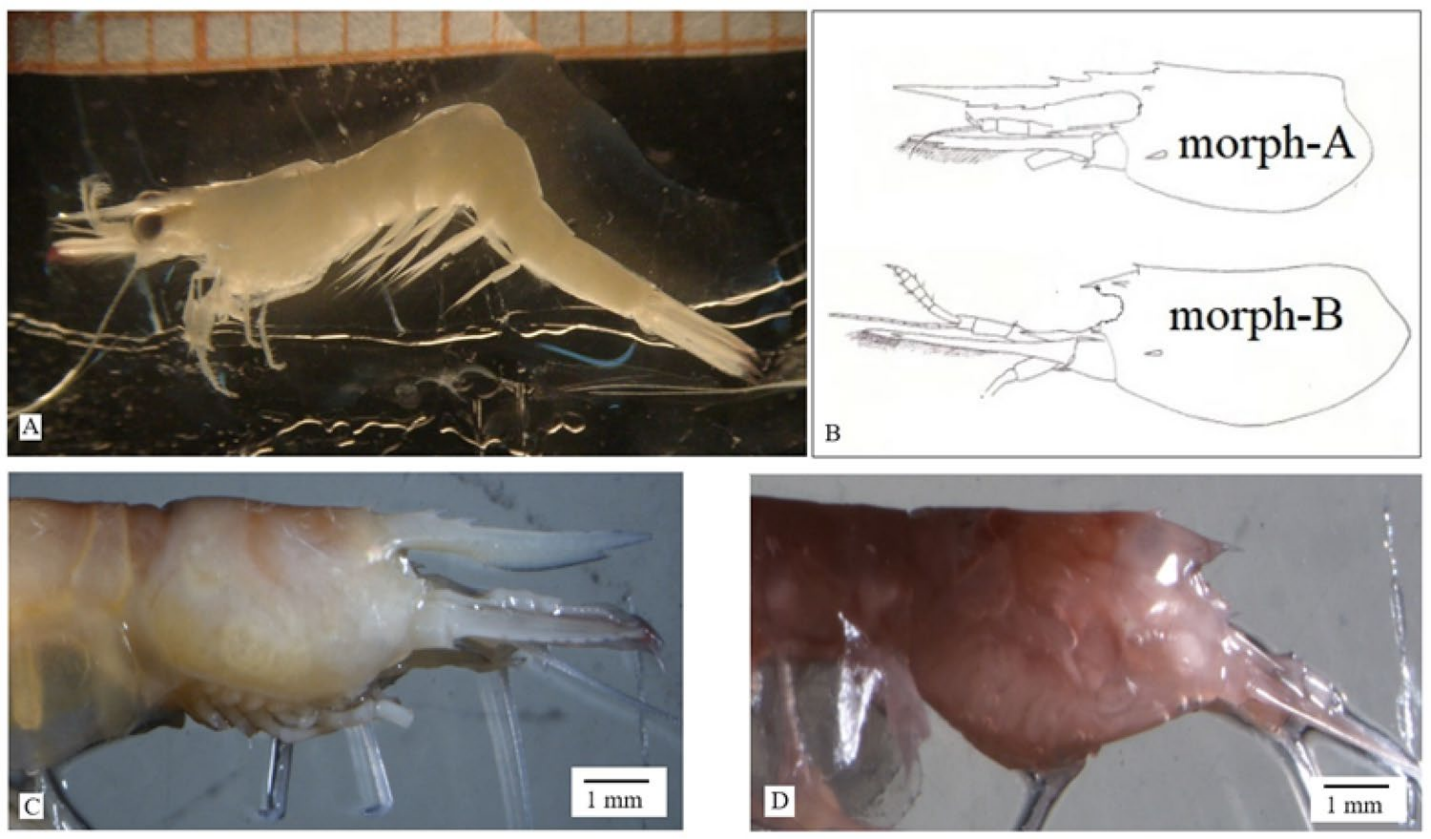
(**A**) *Hippolyte sapphica* morph-A under stereomicroscope. (**B**) Schematic representation of carapace and rostrum of *H. sapphica* morphotypes (modified by d’Udekem d’Acoz, 1996). (**C**) Ovigerous female of *H. sapphica* morph-A. (**D**) Ovigerous female of *H. sapphica* morph-B.

The remarkable phenomenon of the intraspecies variability is quite widespread in marine shrimps and produces polymorphisms often related to shape, color, behavior, and function^1–10^. Documented rostral shape variability for the genus *Hippolyte* is restricted to eleven of the forty species^11,12^. Usually, species rostral variability is recorded between sexes (sexual dimorphism)^13,14^ or in the same sex as a continuous situation with intermediate rostral forms, with slight differences in rostral dentition/inclination^15^. The only sharp rostral dimorphism, with two distinct conspecific morphotypes, appears in *Hippolyte sapphica*^4,12,16^, with morph-A bearing a long, dentate and morph-B a short, toothless rostrum. Morph-B rostra are very short, juvenile-like structures, which confirm the neotenic characters in hippolytids and are referred as “rostral loss” condition^7^ (Fig. 1).

The parsimonious hypothesis that “rostral loss” may be attributed to a single pair of alleles, with a complete dominance of allele b expressed in morph-B shrimps, confirmed by^6^. Additionally, it seems that rostral geometric morphometric analysis reveales the existence of two distinct clusters in morph-B, which correspond to the homozygous and heterozygous state of the gene site (BB and BA) that controls the species phenology^12^. The assumption that morpho-B females develop some compensatory morphological traits, such as enlargement of some body structures (abdominal somites, scaphocerite and telson), in order to improve their hydrodynamic stability, and compete the micro-evolutionary disadvantage of the “rostral loss” it is also confirmed^7^.

The “rostral loss” condition in *H. sapphica* animal model seems to induce life history events on the shrimp’s morphotypes in a complex manner. Although the appearance of sex appendages in morphotypes A and B is performed simultaneously, they were consistently developed later in mature females than in males^6^. Furthermore, morph-B reduces the viability and probability of egg-bearing among large females, demonstrates rapid sexual differentiation, and shows higher propensity to become males^6^. Recent study on the reproduction and eggs’ metrics of *H. sapphica* morphotypes showed that this intraspecific rostral dimorphic system regulates selectively an unequal growth investment and possible acts on sex ratios and reproductive energy investment of the species’ morphs^17^.

Based on the above findings, the present contribution aims to detect and confirm specific mechanisms arisen in population dynamics and life history of *H. sapphica* induced by the “rostral loss” condition. Our objectives are to study thoroughly the sex ratio, the reproductive traits and the population dynamics of the species’ morphotypes and to compare a mixed population (morph-A and morph-B) with a pure, unmixed (morph-A) one of *H. sapphica* throughout a year.

## Results

### Mixed population of *Hippolyte sapphica*

#### Annual dynamics of sex ratios

**Figure 2:**
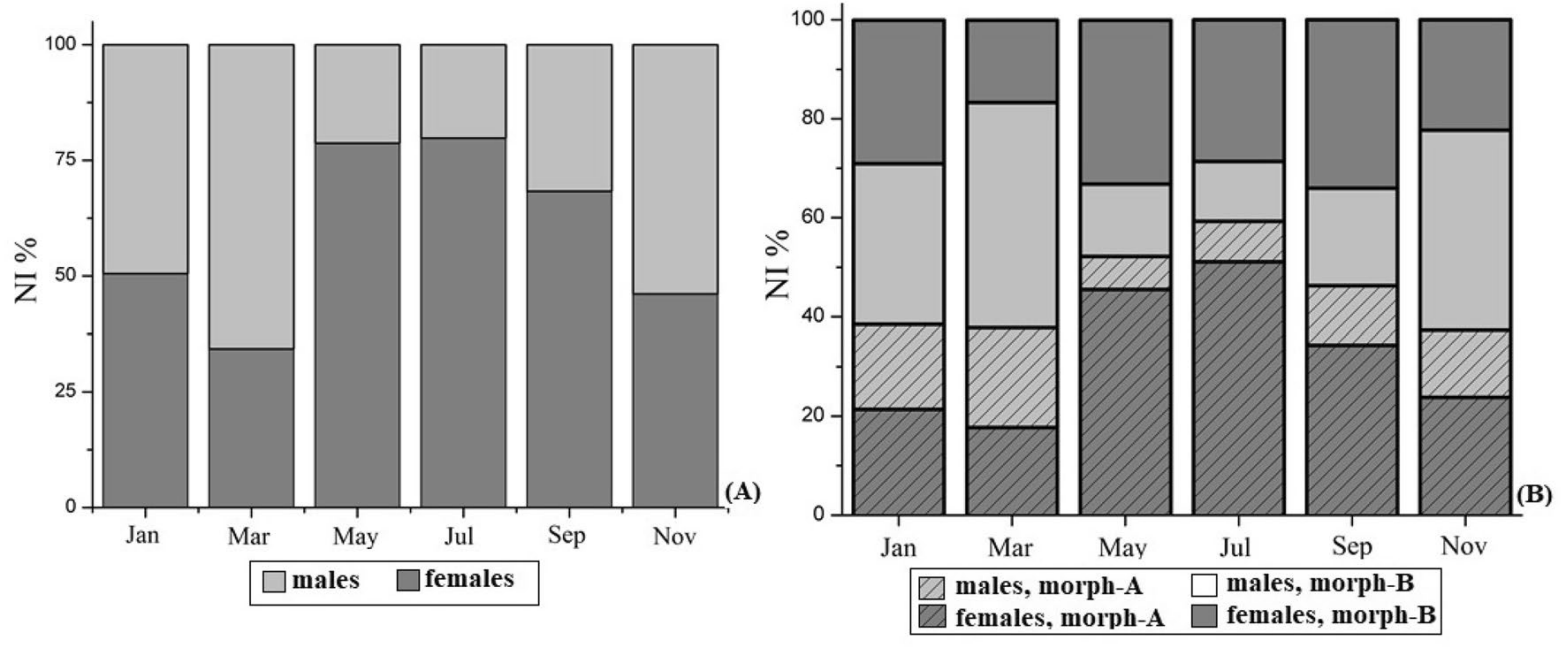
(**A**) Overall bimonthly sex ratio of *H. sapphica.* (**B**) Morph-A and morph-B bimonthly sex ratio of *H. sapphica*. NI%: percentage of the number of individuals.

The sex ratios dynamics of *H. sapphica* were studied in a total of 3020 individuals, 1199 males and 1821 females. The overall sex ratio (males to females) was estimated to be 1:1.52 and found in favor of the females (χ^2^ = 128.1, *P* < 0.001). The seasonal overall sex ratio shows that from November to March the species population is male dominated with its higher percentage in March (χ^2^ = 89.3, *P* < 0.001, males: 65.67% and females: 34.33%), while females dominate from May to September with its higher percentage in July (χ^2^ = 355.3, *P* < 0.001, males: 20.24% and females: 79.76%) (Fig. 2A). The seasonal sex ratio of *H. sapphica* given by morphotype (Fig. 2B) showed that morph-A (long rostrum) found to dominate during May and July {expB = 1.671 (1.076–2.595)}, while morph-B (short rostrum) dominated from September to March.

Considering morph-B to be the reference category, both sex and month factors found to be highly significant (*P* < 0.001). The probability of morph-A among males was lower than among females {expB = 0.312 (0.170–0.572)}. The interaction of the two factors (sex and month) was not significant (*P* = 0.148). After Bonferroni adjustment, the proportions of morphs among males were not significantly different through the seasons, whereas among females, May and July were still significantly different from all other months. Due to the emerging relationship between morph and sex, we observe that the male population declines, when morph-B individuals generally decline too (logistic regression, *P* = 0.05).

#### Reproductive traits

In total, 1626 females counted during the breeding period, which lasts seven months from March to September. The overall proportion of ovigerous females was significantly affected by month, being higher in March (61.22%) with September following (57.68%) (Pearson chi-square *P* < 0.001) (Table 1).

**Table 1:** Monthly ratio and % percentage of the ovigerous females for the breeding period.

Month	NI			Ratio	χ^2^	% Ratio OF/NOF
	OF	NOF	Total			
March	191	121	312	1.58:1	15.70	61.22/38.78
May	107	140	247	0.76:1	4.41	43.32/56.68
July	245	555	800	0.44:1	120.13	30.62/69.38
September	154	113	267	1.36:1	6.29	57.67/42.33
Total	697	929	1626	0.75:1	33.10	42.86/57.14

*NI* Number of individuals, *OF* Ovigerous females, *NOF* Non-ovigerous females.

**Figure 3:**
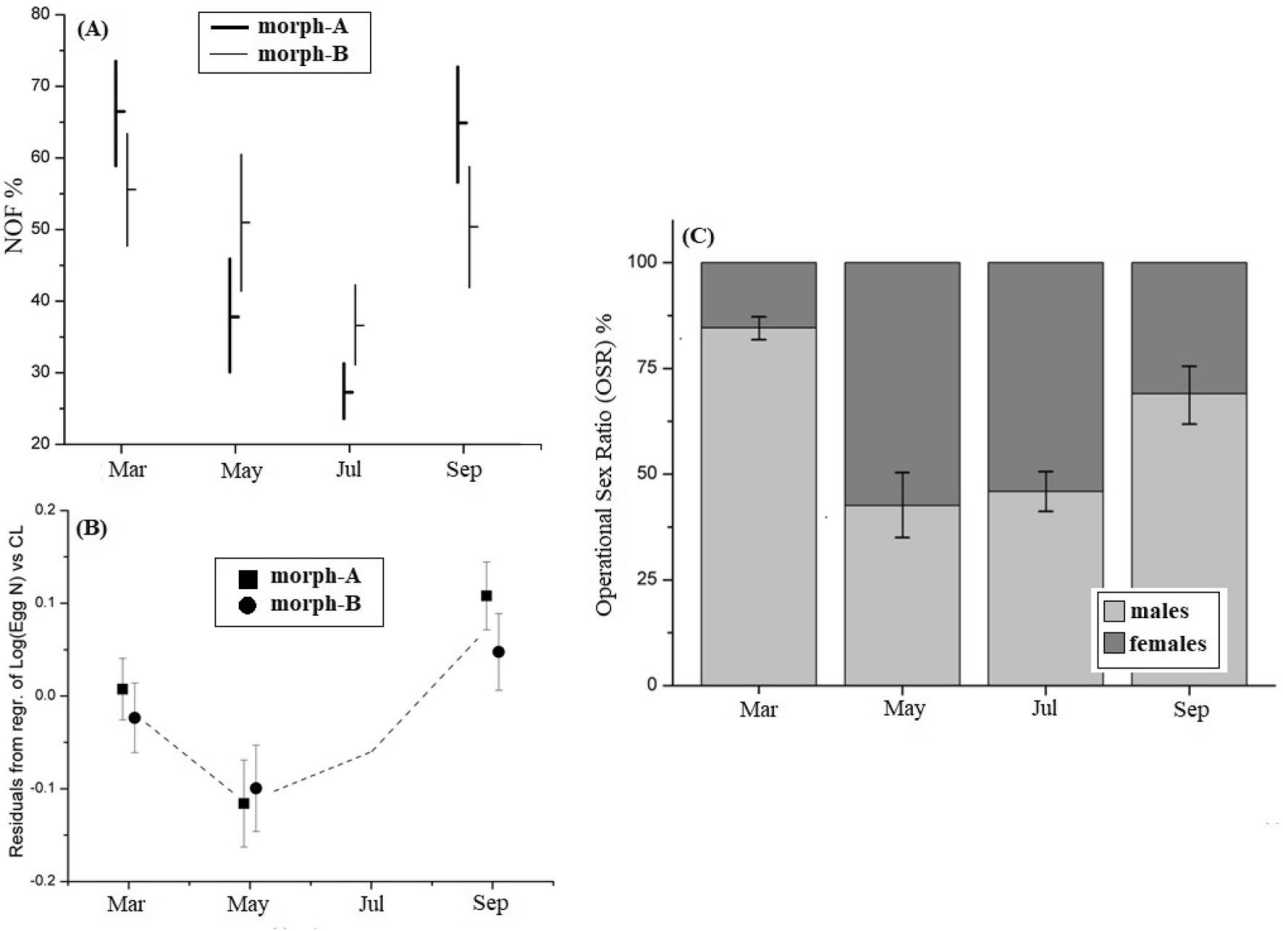
(**A**) Percentage % of *H. sapphica* ovigerous females to the total number of non-ovigerous females (horizontal lines), with their confidence intervals (vertical lines), calculated by binomial test. NOF: number of ovigerous females. (**B**) Mean values of the unstandardized residuals resulting from the regression of Log(EggN) *vs* carapace length (CL) for both morphotypes. (**C**) Operational sex ratio (OSR) of *H. sapphica* mixed population for the breeding period. Error bars represent the 95% CI for males.

Between morph-A and morph-B, the proportion of ovigerous females was different, following the same pattern (Fig. 3A). Ovigerous females were more abundant within morph-A in March and September (Mantel–Haenszel statistic *P* = 0.497; Breslow–Day *P* < 0.001), which means that there doesn’t exist an overall pattern, but the proportions change in opposite direction for the two morphotypes across the seasons (Fig. 3A). It is obvious that morph-A females seem to mature more quickly and give progenies earlier than those from the morph-B (Fig. 3A). The study of fecundity in the mixed population of *H. sapphica* showed that the egg number was strongly dependent on carapace length (CL, F: 60.818; *P* < 0.001; Eta^2^: 0.122), but also significantly changed seasonally (CL, F: 2.923; *P*: 0.000; Eta^2^: 0.113). However, the regression coefficients, which reflect the dependence of egg number from the interactions of morph to CL (F: 2.382; *P* = 0.123; Eta^2^: 0.005) and of month to CL (F: 1.074; *P* = 0.342; Eta^2^: 0.005), did not found significantly different. During the breading period, the egg number reached its highest value in September, and the lowest in May for both morphotypes’ individuals (Fig. 3B).

Additionally, for the further understanding of the species’ reproductive traits in the mixed population, the operational sex ratio (OSR) was calculated only for the breeding period. The size maturity for males and females was defined as CL > 1 mm and CL > 2 mm, respectively^6^. The results show that the OSR was not uniform in the breeding period (Pearson chi-square, *P* < 0.001). During the two principal reproductive bursts (March and September), the OSR was male-biased, (Fig. 3C), while in the middle of the breeding period, where the males seem to be slightly less abundant, the OSR was not significantly different from 1:1.

#### Size frequency distribution and length–weight relationships

**Figure 4:**
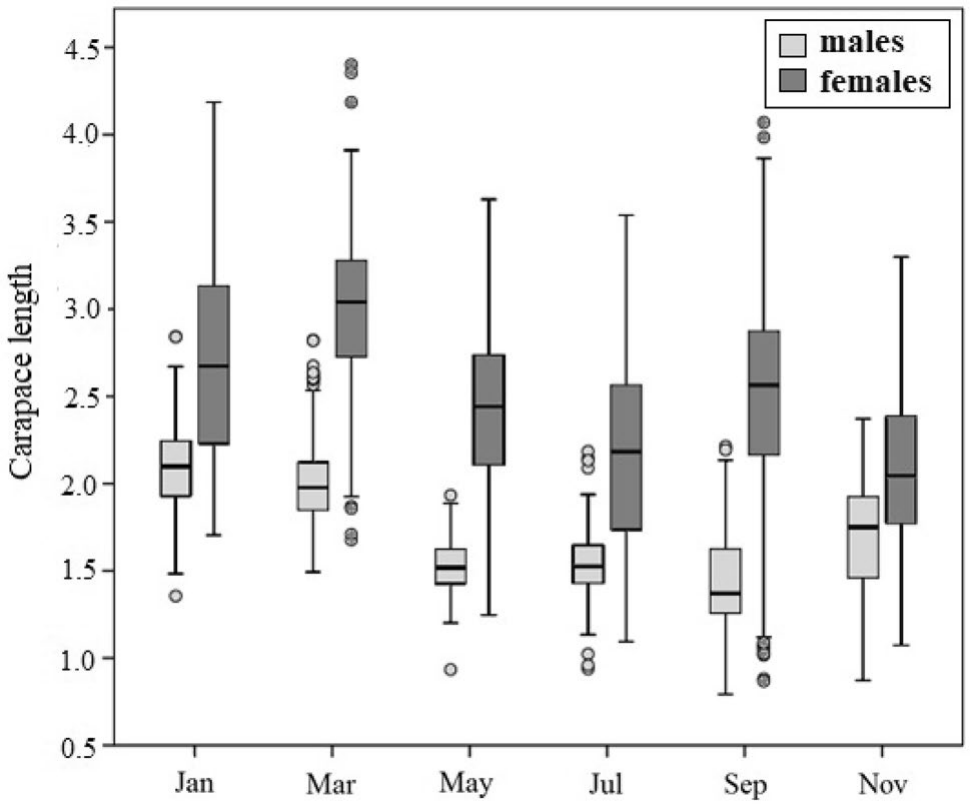
Box plots of seasonal carapace variation of *H. sapphica* in the mixed population given by sex.

The values of CL were significantly different seasonally in both males and females (Kruskal–Wallis, *P* < 0.001; Fig. 4) and the dynamics of the size distribution reveal the differences between the two sexes. More specifically, the period from November to January is characterized by growth because the CL increases (*P* < 0.001) in both sexes. Females continue to grow from January to March, while males present a slightly decrease of CL (*P* < 0.05), which indicates the mortality of the larger males. From March to May there is a sharply decrease of CL for both sexes (*P* < 0.001), the reproduction begins, and the recruitment of the young specimens starts for both sexes, while large ones decline through mortality. During the next two-month period (May–July), only females’ CL continues to decrease (*P* < 0.001) simultaneously with an intense recruitment. From July to September, males’ CL does not increase although its range does, while females’ CL increase along with range (*P* < 0.001) and recruitment. Finally, from September to November, male specimens mainly grow while females demonstrate a sharply decrease of CL (*P* < 0.001), similarly to the one observed in March (Fig. 4).

**Figure 5:**
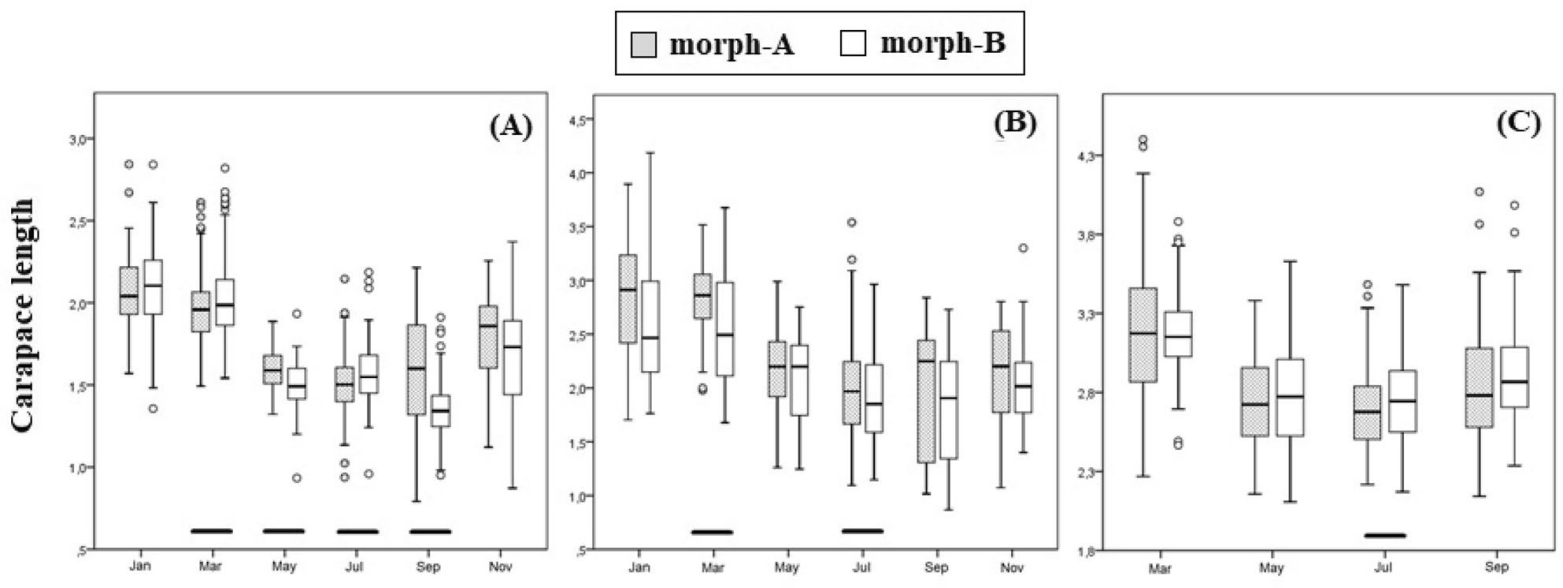
Length frequency distribution of *H. sapphica* mixed population given by morphotype and sex. (**A**): males, (**B**): non-ovigerous females, (**C**): ovigerous females. Horizontal bars indicate the significant difference in carapace length (CL) between morphotypes (Mann–Whitney test).

Our results from the Kruskal–Wallis analysis and the Mann–Whitney test show that the non-ovigerous females of morph-A are growing faster and reach the breeding maturation phase more rapidly than those of morph-B (Table 2, Fig. 5).

**Table 2:** The Mann–Whitney test statistics of carapace length (CL) between morph A and B of the mixed population of *Hippolyte sapphica* split by month and sex.

Month	Sex	CL between forms	Mann–Whitney statistics *P*
January	M		0.915
	NOF	Form A > Form B	0.048
March	M	Form A < Form B	0.008
	NOF	Form A > Form B	0.013
	OF		0.679
May	M	Form A > Form B	0.014
	NOF		0.596
	OF		0.679
July	M	Form A < Form B	0.018
	NOF	Form A > Form B	0.043
	OF	Form A < Form B	0.030
September	M	Form A > Form B	0.000
	NOF		0.074
	OF		0.199

*M* Males, *NOF* Non-ovigerous females, *OF* Ovigerous females.

**Figure 6:**
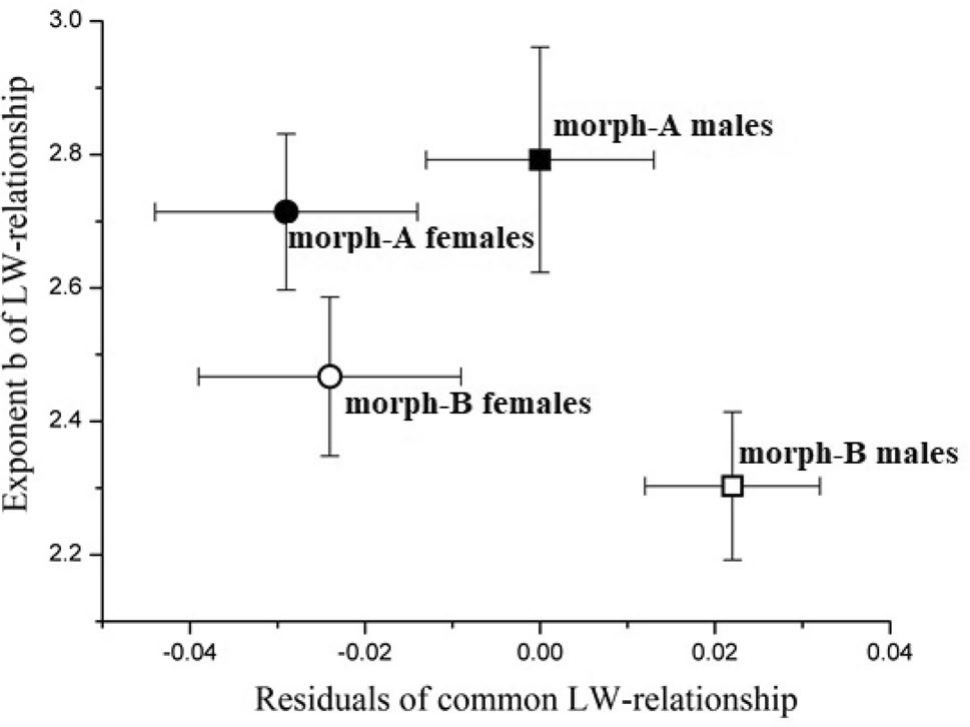
Representation of the negative allometry of *H. sapphica* mixed population estimated by sex and morphotype. Bars represent the 95% of CI.

The length–weight relationships (LWR) were estimated by morphotype and sex (Table 3, Fig. 6). Carapace lengths ranged from 0.79 to 3.89 mm in morph-A and from 0.87 to 4.19 mm in morph-B, while the total weights ranged from 0.001 to 0.180 g and from 0.001 to 0.062 g, respectively (Table 3). Both morphotypes and sexes of *H. sapphica* showed negative allometric growth (b < 3) (Table 3, Fig. 6).

**Table 3:** Estimated parameters of the length–weight relationships of *H. sapphica*, given by morph and sex.

Mo/Sex	NI	CL (mm)	TW (g)	Length–weight relations’ parameters						
		Min–Max	Min–Max	a	95% CI of a	b	95% CI of b	r^2^	*P*	t-test
A/M A/ F	379	0.79–3.37	0.002–0.180	0.305	0.261–0.350	2.792	2.623–2.961	0.855	< 0.001	32.404
	263	0.98–3.89	0.001–0.061	0.268	0.224–0.312	2.714	2.597–2.831	0.939	< 0.001	45.638
B/ M B/ F	660	0.87–4.19	0.001–0.030	0.452	0.421–0.483	2.303	2.192–2.414	0.842	< 0.001	40.748
	260	0.88–3.78	0.004–0.062	0.361	0.318–0.404	2.467	2.348–2.587	0.924	< 0.001	40.589

*NI* Number of individuals, *Mo* Morphotype, *M* Males, *F* Females, *CL* Carapace length, *TW* Total weight.

### Comparison between mixed and unmixed populations of *Hippolyte sapphica*

**Figure 7:**
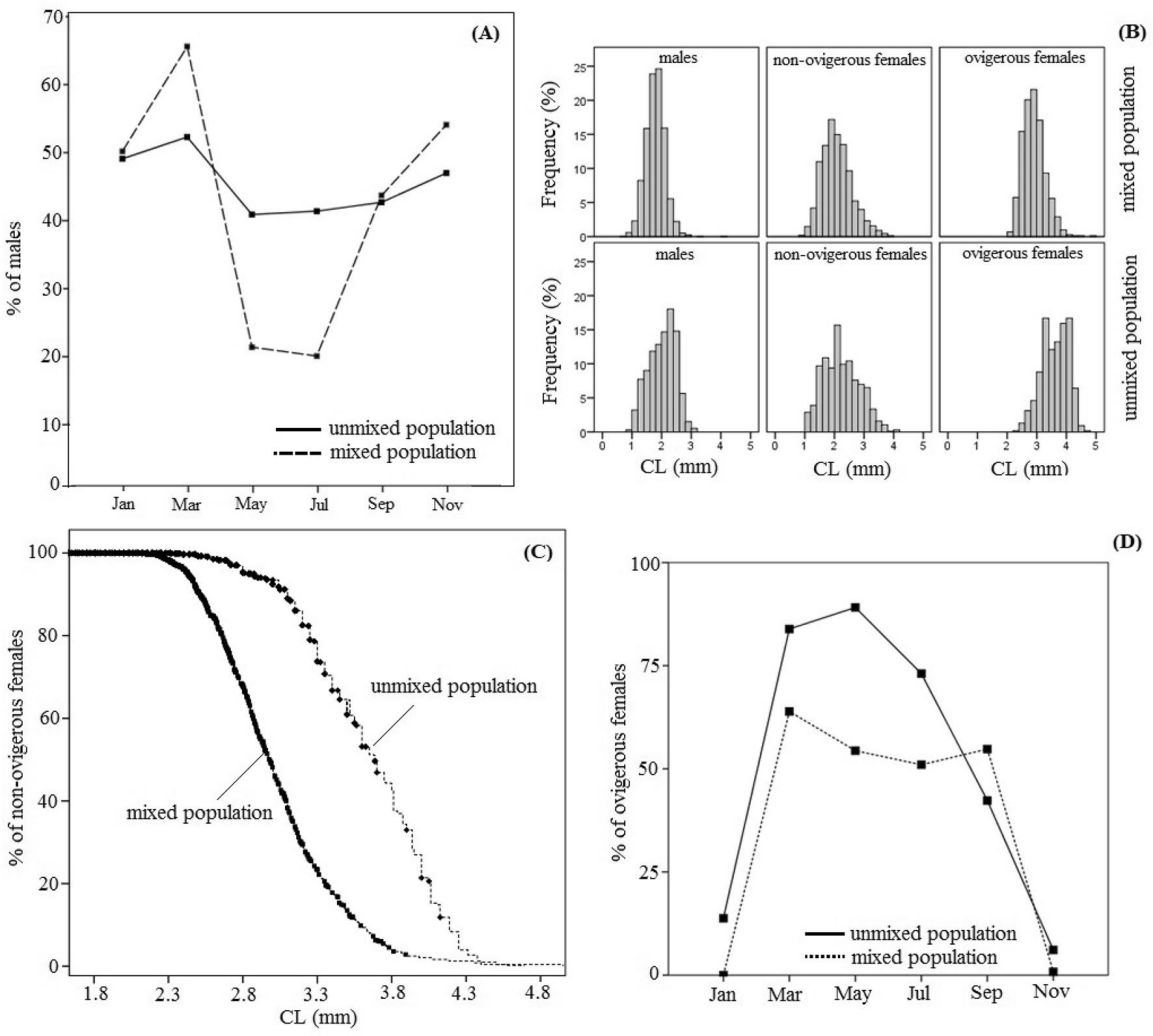
(**A**) Seasonal male % between the mixed (morph-A and morph-B) and unmixed (morph-A) populations of *H. sapphica*. (**B**) Size frequency distribution among the studied biological categories (males, non-ovigerous females and ovigerous females) and between the mixed (Louros River) and unmixed populations (Sagiada Lagoon) of *H. sapphica.* (**C**) Size at maturity of the *H. sapphica* in the mixed (Louros River) and unmixed populations (Sagiada Lagoon). (**D**) Ovigerous females % (in the appropriate range of Carapace Length > 2 mm) given bi-monthly and comparatively for the unmixed and mixed populations of *H. sapphica*.

The overall sex ratio found in favor to the females in the mixed population (from Louros River), but when conditioned for month, the sex ratios were not significantly different (Mantel–Haenszel test; *P* = 0.62), despite the presence of allele b in the mixed population. However, the odds ratios changed significantly in a monthly base (Breslow–Day test, *P* < 0.001). In both mixed and unmixed populations, a typical and statistically significant (*P* < 0.001 and *P* = 0.003) yearly oscillation was present (Fig. 7A). Males found to dominate during the cold season (from November to March) while females during the hot season (from May to July) (Fig. 7D).

The comparison of the size frequency distributions between the mixed and unmixed populations of *H. sapphica* is given in Fig. 7B. Because there were sharp differences of size among the biological categories (males, non-ovigerous females and ovigerous females) the analysis was run for each category separately. Both Population and Month factors were highly significant, along with their interactions (Table 4). In general, carapace lengths found bigger in the unmixed population, in comparison with the mixed one (Fig. 7B). This fact was obvious especially for males and ovigerous females (Fig. 7B) since the non-ovigerous females were still in the process of development. Both males and ovigerous females reached their pick in January-March (data not shown), therefore, the feeding activity was not significantly dropped down during winter. This difference was not due to the presence of morph-B in the mixed population as the two morphotypes were not different in this regard (*P* = 0.8, data not shown).

**Table 4:** ANOVA calculations for Carapace Length *versus* Population & Month factors, layered by biological category (males, non-ovigerous females, ovigerous females).

Biological category	Source	Type III sum of squares	df	F	Sig	Partial Eta^2^
Males	Corrected model	255.127^a^	11	309.250	< 0.001	0.525
	Intercept	6234.075	1	83,122.503	< 0.001	0.964
	Population	43.409	1	578.799	< 0.001	0.158
	Month	177.628	5	473.682	< 0.001	0.435
	Population*Month	46.117	5	122.981	< 0.001	0.166
	Error	231.071	3081			
Non-ovigerous females	Corrected model	247.875^b^	11	107.582	< 0.001	0.311
	Intercept	4450.990	1	21,249.811	< 0.001	0.890
	Population	6.335	1	30.246	< 0.001	0.011
	Month	202.199	5	193.066	< 0.001	0.269
	Population*Month	25.558	5	24.403	< 0.001	0.044
	Error	549.205	2622			
Ovigerous females	Corrected model	242.109^c^	10	249.104	< 0.001	0.663
	Intercept	952.286	1	9798.001	< 0.001	0.885
	Population	9.154	1	94.184	< 0.001	0.069
	Month	76.430	5	157.276	< 0.001	0.383
	Population*Month	23.880	4	61.426	< 0.001	0.162
	Error	123.142	1267			

^a^R^2^ = 0.525 (Adjusted R^2^ = 0.523).

^b^R^2^ = 0.311 (Adjusted R^2^ = 0.308).

^c^R^2^ = 0.663 (Adjusted R^2^ = 0.660).

**Figure 8:**
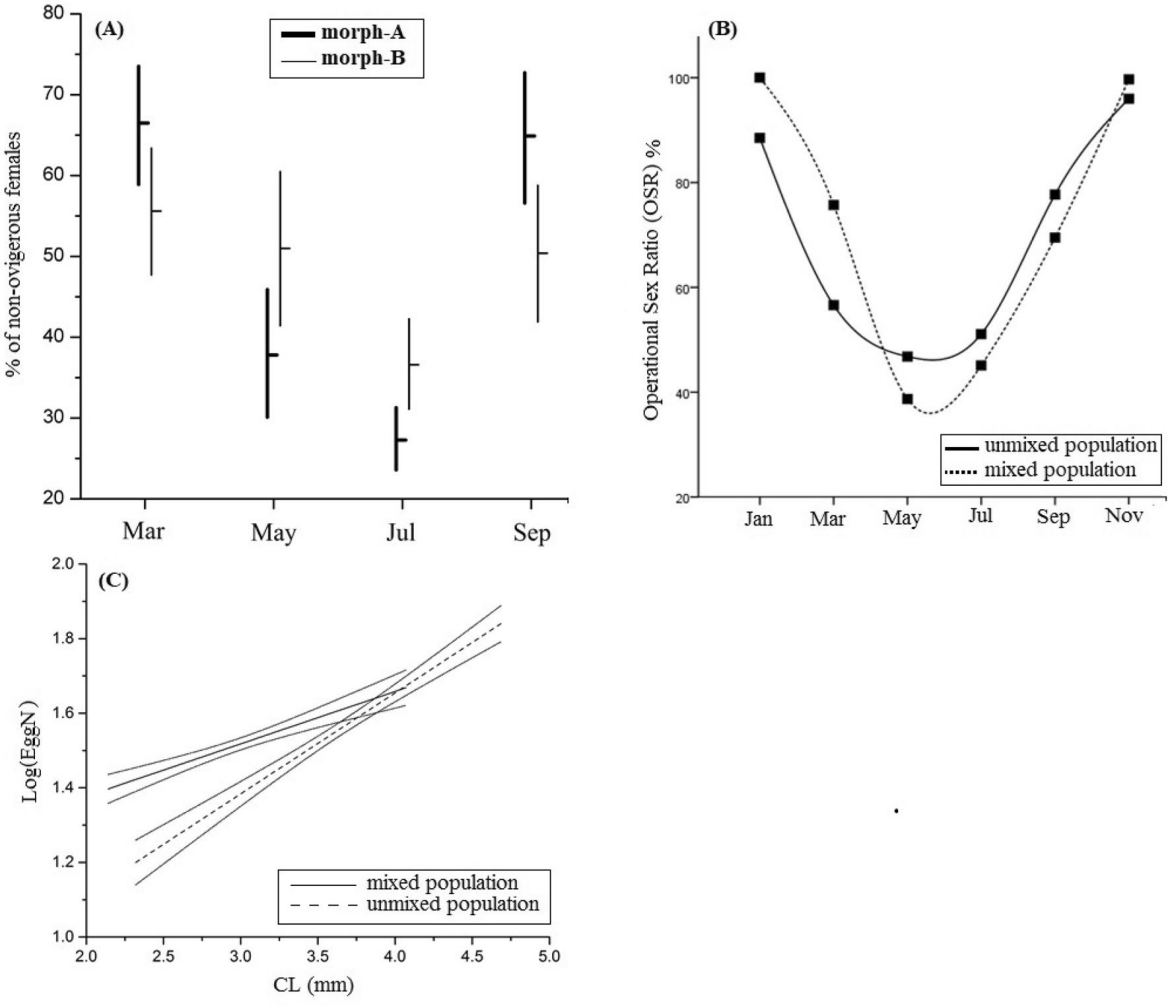
(**A**) Non-ovigerous females % (horizontal lines) of *H. sapphica* in the range of maturity (Carapace Length > 2 mm) with their confidence intervals (vertical lines) calculated by binomial test. (**B**) The operational sex ratios of the *H. sapphica* in the mixed and unmixed populations. (**C**) Fecundity (logEggN) of *H. sapphica* mixed and unmixed populations given by carapace length (CL). logEggN = log of the number of eggs.

The median size for maturity, however, was significantly smaller in the mixed (2.98 mm ± 0.04) than in the unmixed population (3.69 mm ± 0.05) (binary logistic regression and Kaplan–Meier analysis). The S-curves for the two studied populations are shown in Fig. 7C. The percentage of the egg-bearing specimens of *H. sapphica* in the appropriate range of carapace length (CL > 2 mm) found significantly higher in the unmixed population (Mantel–Haenszel test: *P* < 0.001) (Fig. 7D). Furthermore, this relationship was not uniform across the different months (Breslow–Day test: *P* < 0.001, Fig. 7D). The egg production for the unmixed population did not stop even during the winter months and its intensity was higher in comparison to the mixed one. The only exception was during September, when the situation was reversed (*P* = 0.002). For the mixed population, the presence of morph-B, gave lower egg production during the period September–March and generally lower number of females (Fig. 8A). The operational sex ratio for the two studied populations was calculated and presented in Fig. 8B. More specifically, the percentage of the males found higher in the mixed population from January to March, while the females’ percentage recorded higher during the summer and autumn period. Overall, the sex-ratio changed significantly amongst the months (Breslow–Day; *P* < 0.001) and, paradoxically, the conditioned by month operational sex ratio found higher for the mixed population (Mantel–Haenszel test; *P* = 0.036). Despite the male-oriented “influence” of the morph-B (presence of allele b in the mixed population), the mixed population seems to hyper compensate its effect. The fecundity was comparatively investigated and the ANCOVA results are presented in Table 5 and Fig. 8C. Both population and its interaction with carapace length found significant. Overall, the females’ specimens from the mixed population demonstrated higher number of eggs in comparison with the unmixed population, with this difference being prominent in the small specimens (Fig. 8C).

**Table 5:** ANCOVA calculations for fecundity (logEggN) of *H. sapphica* with population as factor and carapace length (CL) as covariate.

Source	Type III Sum of Squares	df	Mean Square	F	Sig	Partial Eta^2^
Corrected model	6.970^a^	3	2.323	71.818	< 0.001	0.203
Intercept	8.700	1	8.700	268.912	< 0.001	0.241
Population	0.859	1	0.859	26.566	< 0.001	0.030
CL (mm)	5.945	1	5.945	183.763	< 0.001	0.178
Population* CL (mm)	0.595	1	0.595	18.376	< 0.001	0.021
Error	27.370	846	0.032			
Total	2039.636	850				
Corrected Total	34.340	849				

EggN, Number of Eggs.

^a^R^2^ = 0.203 (Adjusted R^2^ = 0.200).

## Discussion

Demography and reproductive traits of *H. sapphica* morphotypes revealed some interesting recordings in the different levels of the species bionomic characteristics. The overall sex ratio found to have a predominance of females, and as a result agrees with the sexual ratios of the gonochoric *H. obliquimanus* ^18^, as well as with the sex proportions of *H. obliquimanus* morphotype H^19^. Additionally, the seasonal sex ratio of *H. sapphica* morphotypes (Fig. 2B) revealed an emerging relationship between morphotype and sex: the probability of morph-A among the male individuals found lower than among the female ones, resulting to a decline of the male population along with a general decline of morph-B individuals too. In other words, the rostral state of being long (morph-A) or short (morph-B)} may be regarded as a sex-related marker. If a sex transition from *H. sapphica* males to females occurs during winter, then the morph-A/B ratio among females should be shifted in March towards the state B, in comparison with November (because most of the male individuals are B). However, nothing similar happens. The proportion of morph-A amongst females found exactly the same (51.7 in November and 51.8 in March, *P* > 0.99, Fisher exact test). These results indicate that, at least in *H. sapphica*, the sex transition does not occur, and the species is gonochoric. Among the 40 species of the genus *Hippolyte*^12,20–25^ five species have been characterized as protandric hermaphrodites, three species as gonochoric and for the rest the data are incomplete or deficient^17,22,26^. Complete and thorough studies on the sexual systems have been accomplished only for four species (*H. inermis, H. niezabitowskii, H. obliquimanus* and *H. williamsi*)^22,26–36^ emerging that there is a need for more relevant information on this field. The gonochoric status of *H. sapphica* based on the overall balance of sex ratio, as well as on the absence of the any negative allometry of the appendix masculina in large male specimens has been also supported by^6^. This first information on the gonochoric status of the species is amplified by the present data of the sex ratios annual dynamics of *H. sapphica* morphotypes.

The reproductive period of *H. sapphica* lasts all over the year. According to our results, although the ovigerous females from the mixed population of the species have been recorded only from March to September, the ones from the unmixed population have been sampled throughout the seasons, and during the winter months too (Fig. 7D). The two only species of the genus, which demonstrate extended yearly breeding period are *H. varians* and *H. zostericola*, while the majority of the species breed mainly during the spring–summer or spring–autumn periods^11,22,37^. The comparison, within the breeding period, of the ovigerous females’ percentage between the two morphotypes showed a seasonal succession (Fig. 3A), which gives a temporal advantage to morph-A individuals, by maturing and giving progenies earlier. Similar types of temporal equilibria within morphs have been also recorded in *H. obliquimanus* morphs’ habitat use^9^. Moreover, significantly different sex ratios in *Penaeus semisulcatus* morphotypes and female domination throughout the year for the banded morphotype have been found by^38^. The species fecundity did not demonstrate any differences between the two morphotypes, a fact which is also confirmed by^17^, who studied fecundity and egg metrics from species populations along its distributional range. According to^17^ only the reproductive output (RO = Brood Dry Weight/Female Dry Weight), which indicates the energy investment of the reproductive females, found lower in morph-B ovigerous females. In the present study, the operational sex ratio (OSR) showed variations during the breeding period and found male-biased (Fig. 2C), a fact that indicates a competition of males for available females. The presence of morph-B may exacerbate the sexual competition for females during the two reproductive bursts, making this factor especially important for *H. sapphica* intraspecific interactions.

The annual size frequency distribution of *H. sapphica* shows that the males’ spring recruitment lasts until May, while the females’ one is more prolonged and last until July. The second wave of recruitment happens earlier in males in late August, while in females it occurs from September to November, accompanied by the mortality of the large specimens. Because of this observed asynchrony, some large males die before the reproduction period, which starts in March. Between the two morphotypes, we recorded that the A-non-ovigerous females are growing faster in comparison with those of morph-B. Thus, this intraspecific rostral dimorphic system offers viable advantages in morph-A which are connected to the life strategy of the species. Similar notable differentiations in population dynamics and size-frequency distributions have been shown in *Penaeus semisulcatus* morphotypes^38^ and through contrasting life-history strategies of two sympatric *Palaemon* shrimps from shallow marine habitats^39^. It seems that in coastal dynamic ecosystems, some population parameters of the r-selected shrimps could be modified, in an inter- and intra-specific level, to reach appropriate equilibria, intrinsic to the species’ life history.

The comparison of the mixed to unmixed populations of *H. sapphica* reveals some specific adaptations of the two morphotypes in accordance with the population structure, dynamics, and reproduction. First, regarding the sex ratios, the profound drop of the male proportion in in the mixed population during May–July may be regarded as a compensatory adaptation to a possible preferential spawning. In both mixed and unmixed *H. sapphica* populations, we found a typical, yearly oscillation (Fig. 7A), with males’ dominance during the cold season and females’ one during the hot season (Fig. 7D). The size frequency distributions comparisons revealed that the unmixed population from Sagiada Lagoon demonstrated bigger carapace sizes in general, a fact that could be related mainly to the predation pressure rather than the trophic conditions of the lagoonal ecosystem. More specifically, the mixed population was sampled from Louros estuary habitat from a channel connected with Tsoukalio Lagoon, which supports extensive culture of fish and is characterized by wide salinity/dissolved oxygen ranges (11.6–28.9/DO: 6.0–10.7 mg/L) and chl-a concentrations from 0.2 to 6.5 ug/L^40^. The unmixed population of *H. sapphica* sampled from Sagiada lagoon near the connection with Kalamas estuary, where the typical values of chl-a were ranged between 0.54 and 6.14 mg/m^3^^41^.

The median size for maturity found significantly higher in the unmixed population (Fig. 6C), a result that may be considered as an adaptation for the higher body growth. Ovigerous females found also bigger in all unmixed populations from the Aegean sites^17^, reaching their maximum values in stations with favorable environmental requirements such as food adequacy, well-established *Cymodocea nodosa* meadows, sheltered and shallow bays. Other hippolytid interpopulation studies tried to correlate the mean size of ovigerous females with latitude^35^, in order to reveal possible geographical trends in the reproductive ecology of the species. Nevertheless, only the egg metrics (embryo number, embryo volume and reproductive output) found to be affected by latitude^35^. In our study, the unmixed population is characterized by intense egg production in comparison to the mixed one, which last all over the year. The presence of morph-B in the mixed population, had as a result lower egg production and generally lower number of females (Fig. 8A). The operational sex ratio for the two studied populations (Fig. 8B) is seasonally affected. Males’ percentage of was higher in the mixed population from January to March, while the females’ percentage recorded higher during summer and autumn periods. Paradoxically, the conditioned by month operational sex ratio found higher for the mixed population (Mantel–Haenszel test; *P* = 0.036). This means that despite the presence of allele b in the mixed population, which supports a male-oriented “influence”, *H. sapphica* mixed population seems to hyper compensate its effect. Finally, fecundity, when studied comparatively for the two species populations (Fig. 8C), found to be higher in the mixed one, with this difference being prominent for the small ovigerous female specimens.

## Conclusions

Intra-species shape and color morphotypes in marine ecosystems usually reveal specializations and adaptations in the level of their life history, aiming at the utilization of all available sources and habitats. Among them, shape polymorphisms are rather rare in comparison to the color ones, and they attribute a special importance to the biological models that display them. Within the range of shrimps’ rostral diversity, the most distinct, loud examples which have been observed in nature are the “Pinocchio-shrimp effect” related to the rostral length of freshwater atyids, the sexual dimorphic rostral shortening in aristeids, and the “rostral loss” in hippolytids. The results we obtained in the present study shed light on life history adaptations and reproductive strategies of *H. sapphica* morphotypes’ biological model.

The study of the seasonal sex ratios in *H. sapphica* morphotypes revealed that this rostral dimorphic system of being long (morph-A) or short (morph-B) is a sex-related marker since the probability of morph-A among the male individuals found lower, resulting to a male population decline and to a general decline of morph-B individuals. Our results indicate that there is no sex transition in *H. sapphica* and support the hypothesis of a gonochoric sexual system. In the mixed populations of the species, both size frequency distributions and seasonal sex ratios showed that morph-A females seem to mature more quickly and give progenies earlier than those from the morph-B. In both mixed and unmixed populations, there is a male dominance during the cold season and a female one in the hot season, which means that the presence of allele b in the species populations does not affect the general pattern of annual sex ratio, exacerbating however the variation on seasonal basis. The “rostral loss” condition seems to offer viable advantages in morph-A individuals which grow faster, have higher probability to become females and increase their proportions during summer, especially in July. Additionally, although morph-B presence had as a result lower egg production and higher males’ percentage in a seasonal base (from January to March), *H. sapphica* populations seems to hyper compensate this shift through fecundity, which found higher in the mixed populations and through the general contribution of morph-A females in size frequency distributions equilibria.

## Methods

### Field samplings and laboratory assessment

For the study of the mixed population (morph-A and morph-B) shrimp samples were collected in a bi-monthly base from Louros River estuary (NW Greece, 39° 13′ 961″ N, 020° 45′ 971″ E). Additionally, a pure, unmixed morph-A population of *H. sapphica* was monitored in a bimonthly base from Sagiada Lagoon (NW Greece, 39°62′ 605″ N, 020° 18′ 105″ E). All individuals were collected by means of a hand net (frame: 30 cm × 35 cm, mesh size: 2 mm), and additionally of a zooplankton net (mesh size 200 μm) to ensure that all size classes were adequately represented. In the field, the sample was preserved in 4% formaldehyde solution. In the laboratory, the species was identified by specialized keys^11,21^, through stereo-microscopic inspection of (a) the absence of teeth on the first article of antennular peduncle, (b) the presence of postrostral tooth, (c) the shorter length of the outer antennular flagellum in comparison to the inner one, (d) the position of the hepatic spine on carapace. Morphotypes were separated according to their rostral morphology^11,12^ and sexes (males/females) were identified by the presence/absence of the appendix masculina on the second pleopod, respectively. All individuals first photographed, weighed by digital assay balance (± 0.1 mg) and CLs measured with the image analysis system ZEN 2012. Brood chamber was dissected from each ovigerous female, and the total number of eggs was measured to evaluate absolute fecundity.

### Statistical analyses

The overall, the seasonal and the operational sex ratios (OSR) between the morph-A and B were compared using Pearson Chi-square test. OSR is the ratio of the sexually competing, mature males to the sexually mature but not egg-bearing yet females. Logistic regression with the morphotype as dependent variable and sex and month as factors was used to estimate the variability of the morphotype ratios between sexes and amongst the seasons. Bonferroni adjustment was applied to pairwise comparisons. The seasonal variation of the proportion of ovigerous female specimens (amongst females in general and between morphotypes) was studied using χ2 test, as well as Mantel–Haenszel and Breslow–Day tests. Confidence intervals for proportions were calculated by binomial test. ANCOVA was applied for the study of *H. sapphica* mixed population fecundity with the number of eggs as dependent variable, morphotype and month as fixed factors and CL as covariate. Seasonal variability of CL in both sexes was studied using Kruskal–Wallis test due to the distribution’s non-normality. Pairwise comparisons were done with Bonferroni correction. Differences between morphotypes, split by month and sex were estimated by Mann–Whitney test. The LWR was assessed by ANCOVA, with weight as dependent variable, sex and morphotype as factors, and CL as covariate. The sex ratio, proportion of the ovigerous females among females and the OSRs for two studied populations (mixed and unmixed), conditioned for season, were estimated by Mantel–Haenszel and Breslow–Day tests. The median size for maturity between mixed and unmixed populations of the species was compared by binary logistic regression (population as factor and CL as covariate) and estimated by Kaplan–Meier analysis. Fecundity in the two studied populations was compared by ANCOVA, having the population as factor and CL as covariate.

## Data Availability

All collected material is available and deposited in Fisheries Research Institute (Hellenic Agricultural Organization ‘Demeter’, Kavala) under the supervision of Dr Chryssa Anastasiadou (anastasiadou@inale.gr) and all analysis dataset is also available by Dr Roman Liasko (rliasko@uoi.gr).

## References

[1] Kuris AM, Ra’anan Z, Sagi A, Cohen D (1987). Morphotypic differentiation of ale Malaysian giant prawns, *Macrobrachium rosenbergii*. J. Crustac. Biol..

[2] Bauer, R. T. Remarkable Shrimps: Adaptations and Natural History of the Carideans. In: *The Animal Natural History Series.* vol. 7, 1–279 (University of Oklahoma Press, USA) (2004).

[3] Bauer RT (2009). Polymorphism of colour pattern in the caridean shrimps *Heptacarpus pictus* and *H. paludicola*. Mar. Behav. Physiol..

[4] Ntakis A, Anastasiadou Ch, Liasko R, Leonardos I (2010). Larval development of the shrimp *Hippolyte sapphica* d’Udekem d’Acoz, 1993 forma A and B (Decapoda: Caridea: Hippolytidae) reared in the laboratory, confirmation of the conspecific status of the two forms. Zootaxa.

[5] Wortham JL, Van Maurik LN (2012). Morphology and morphotypes of the Hawaiian river shrimp *Macrobrachium grandimanus*. J. Crustac. Biol..

[6] Liasko R, Anastasiadou Ch, Ntakis A, Leonardos ID (2015). How a sharp rostral dimorphism affects the life history, population structure and adaptability of a small shrimp: the case study of *Hippolyte sapphica*. Mar. Ecol..

[7] Liasko R, Anastasiadou Ch, Ntakis A (2018). Eco-morphological consequences of the “rostal loss” in the intertidal marine shrimp *Hippolyte sapphica* morphotypes. J. Mar. Biol. Assoc. U. K..

[8] Duarte RC, Flores AAV (2016). Morph-specific habitat and sex distribution in the caridean shrimp *Hippolyte obliquimanus*. J. Mar. Biol. Assoc. U. K..

[9] Duarte RC, Stevens M, Flores AAV (2016). Shape, colour plasticity, and habitat use indicate morph-specific camouflage strategies in a marine shrimp. Evol. Biol..

[10] Duarte RC, Flores AAV, Vinagre C, Leal MC (2017). Habitat dependent niche partitioning between colour morphs of the algal dwelling shrimp *Hippolyte obliquimanus*. Mar. Biol..

[11] D’Udekem d’Acoz C (1996). The genus *Hippolyte* Leach, 1814 (Crustacea, Decapoda, Caridea: Hippolytidae) in the East Atlantic Ocean and the Mediterranean Sea, with a checklist of all species in the genus. Zool. Verh. Leiden.

[12] Anastasiadou C, Liasko R, Kallianiotis AA, Leonardos I (2022). Rostral geometric morphometrics in a hippolytid shrimp: Are there elements that reflect the homozygous/heterozygous state of its morphotypes?. J. Mar. Sci. Eng..

[13] Sardà F, Demestre M (1989). Shortening of the rostrum and rostral variability in *Aristeus antennatus* (Risso, 1816) (Decapoda: Aristeidae). J. Crustac. Biol..

[14] Christodoulou M, Anastasiadou C (2017). Sexual dimorphism in the shrimp genus *Atyaephyra* De Brito, 1867 (Caridea: Atyidae): The case study of *Atyaephyra thyamisensis* Christodoulou, Antoniou, Magoulas & Koukouras, 2012. J. Crustac. Biol..

[15] De Mazancourt V, Marquet G, Keith PH (2017). The “Pinocchio-shrimp effect”: First evidence of variation in rostrum length with the environment in Caridina H. Milne-Edwards, 1837 (Decapoda: Caridea: Atyidae). J. Crustac. Biol..

[16] D’Udekem d’Acoz C (1993). Description d’ une nouvelle crevette de l’ île de Lesbos: *Hippolyte sapphica* sp. Nov. (Crustacea, Decapoda, Caridea: Hippolytidae). Belg. J. Zool..

[17] Anastasiadou C (2022). Reproductive variability in hippolytid shrimp shape morphotypes. J. Zool. Syst. Evolut. Res..

[18] Terossi M, Mantelatto FL (2010). Sexual ratio, reproductive period and seasonal variation of the gonochoric shrimp *Hippolyte obliquimanus* (Caridea: Hippolytidae). Mar. Biol. Res..

[19] Duarte RC, Flores AAV (2016). Morph-specific habitat and sex distribution in the caridean shrimp *Hippolyte obliquimanus*. J. Mar. Biol. Ass. U. K..

[20] D’Udekem d’Acoz C (1999). Inventaire et distribution des Crustacés Décapodes de l’ Antlantique nord-oriental, de la Méditerranée et des eaux continentales adjacentes au nord de 25 N. Patrims. Nat..

[21] D’Udekem d’Acoz C (2007). New records of Atlantic *Hippolyte*, with the description of two new species, and a key to all Atlantic and Mediterranean species (Crustacea, Decapoda, Caridea). Zoosystema.

[22] Espinoza-Fuenzalida NL, Thiel M, Dupre E, Baeza A (2008). Is *Hippolyte williamsi* gonochoric or hermaphroditic? A multi-approach study and a review of sexual systems in *Hippolyte* shrimps. Mar. Biol..

[23] Terossi M, De Grave S, Mantelatto FL (2017). Global biogeography, cryptic species and systematic issues in the shrimp genus *Hippolyte* Leach, 1814 (Decapoda: Caridea: Hippolytidae) by multimarker analyses. Sci. Rep..

[24] Gan Z, Li X (2017). A new species of the genus *Hippolyte* (Decapoda: Caridea: Hippolytidae) from South China Sea and Singapore. Zootaxa.

[25] Fransen CHJM, De Grave S (2019). Two new species of *Hippolyte* from the Tropical Central and East Atlantic (Crustacea, Decapoda, Caridea). Zootaxa.

[26] Manjón-Cabeza ME, Cobos V, García-Raso JE (2011). The reproductive system of *Hippolyte niezabitowskii* (Decapoda, Caridea). Zoology.

[27] Zupo V (1994). Strategies of sexual inversion in *Hippolyte inermis* Leach (Crustacea, Decapoda) from a Mediterranean seagrass meadow. J. Exp. Mar. Biol. Ecol..

[28] Zupo V (2000). Effect of microalgal food on the sex reversal of *Hippolyte inermis* (Crustacea: Decapoda). Mar. Ecol. Prog. Ser..

[29] Zupo V (2001). Influence of diet on sex differentiation of *Hippolyte inermis* Leach (Decapoda: Natantia) in the field. Hydrobiologia.

[30] Zupo V, Mérillon JM, Ramawat K (2020). Co-evolution of the shrimp *Hippolyte inermis* and the Diatoms *Cocconeis spp*. in *Posidonia oceanica*: sexual adaptations explained by ecological fitting. Co-Evolution of Secondary Metabolites.

[31] Zupo V, Messina P (2007). How do dietary diatoms cause the sex reversal of the shrimp *Hippolyte inermis* Leach (Crustacea, Decapoda). Mar. Biol..

[32] Zupo V, Bottino I (2001). Larval development of decapods crustaceans investigated by confocal microscopy: an application to *Hippolyte inermis* (Natantia). Mar. Biol..

[33] Cobos VV, Díaz JE, García-Rasoand M, Manjón-Cabeza E (2005). Insights on the female reproductive system in *Hippolyte inermis*: Is this species really hermaphroditic?. Invertebr. Biol..

[34] Terossi M, Lopez Greco LS, Mantelatto FL (2008). *Hippolyte obliquimanus* (Decapoda: Caridea: Hippolytidae): A gonochoric or hermaphroditic shrimp species?. Mar. Biol..

[35] Terossi M, Wehrtmann IS, Mantelatto FL (2010). Interpopulation comparison of reproduction of the Atlantic shrimp *Hippolyte obliquimanus* (Caridea: Hippolytidae). J. Crustac. Biol..

[36] Terossi M, Tudge C, Lopez Greco LS, Mantelatto FL (2012). A novel spermatozoan ultrastructure in the shrimp *Hippolyte obliquimanus* Dana, 1852 (Decapoda: Caridea: Hippolytidae). Invertebr. Reprod. Dev..

[37] Romero-Rodríguez J, Román-Contreras R (2013). Population structure and reproduction of the seagrass shrimp *Hippolyte zostericola* (Decapoda: Hippolytidae) at Laguna de Términos, Campeche, Mexico. J. Mar. Biol. Assoc. U. K..

[38] Alizadeh E, Safaie M, Momeni M, Kamrani E (2022). Population dynamics of two morphotypes of green tiger shrimp *Penaeus semisulcatus* de Haan, 1844 in the northern coast of Iran. Indian J. Fish..

[39] Vejan A (2022). Comparative population dynamics of two sympatric *Palaemon* shrimps (*Palaemon adspersus* Rathke, 1836 and *Palaemon elegans* Rathke, 1836) from the Southeast Caspian Sea. Front. Mar. Sci..

[40] Kormas KA, Nikolaidou A, Reizopoulou S (2001). Temporal variations of nutrients, chlorophyll *a*, and particulate matter in three coastal lagoons of Amvrakikos Gulf (Ionian Sea, Greece). Mar. Ecol..

[41] Kagalou I, Leonardos I, Anastasiadou C, Neofytou C (2012). The DPSIR approach for an integrated river management framework. A preliminary application on a Mediiterranean site (Kalamas River-NW Greece). Water Resour. Manag..

